# Editorial: Melatonin in Health and Disease

**DOI:** 10.3389/fendo.2020.613549

**Published:** 2020-11-26

**Authors:** James Olcese, Ralf Jockers

**Affiliations:** ^1^Department of Biomedical Sciences, Florida State University College of Medicine, Tallahassee, FL, United States; ^2^Université de Paris, Institut Cochin, INSERM, CNRS, Paris, France

**Keywords:** circadian rhythms, sleep, metabolism, Alzheimer disease, reproduction, pineal gland, antioxidant, G protein coupled receptor

Melatonin was discovered more than 60 years ago as N-acetyl-5-methoxytryptamine, a derivative of tryptophan that is structurally related to serotonin. Since then, an intense research activity developed around this small molecule to which the authors of this Research Topic made seminal contributions.

The first key question in the field was: “Where does melatonin come from or how is it synthesized?”. Dr. Klein and his colleagues pioneered the field of melatonin synthesis, in particular in the pineal gland, the organ of melatonin synthesis in all vertebrates. A distinctive feature of melatonin is its circadian synthesis pattern with high levels during the dark phase and low levels during the light phase. Rhythmicity of melatonin synthesis is achieved by the circadian master clock located in the suprachiasmatic nuclei (SCN) which is under retinal light control. In the present Research Topic, Dr. Klein and colleagues report the latest advances in characterizing the cell types constituting the pineal gland. They defined nine types in the rat pineal gland based on single-cell RNAseq (Coon et al.). Pinealocytes were estimated to account for about 90% of the cells and the remainders are astrocytes, microglia, vascular, and leptomeningeal cells (VLMCs) and endothelial cells. Among the pinealocytes, alpha and beta types are transcriptionally distinct, the beta type representing 95% of all pinealocytes. The authors speculate about a cooperative behavior between both cell types with alpha-pinealocytes being the most specialized and active melatonin synthesizing cells and beta-pinealocytes supporting alpha cells by providing the melatonin precursor N-Acetylserotonin. This kind of single cell analysis is likely to shape our understanding of pineal cell biology in the future.

The issue of melatonin synthesis is then taken further by Dr. Reiter, one of the pioneers in melatonin biology. The authors ask the question of the very first origins of melatonin that is likely to have occurred before the development of the pineal gland. They are advocating for a bacterial origin of melatonin synthesis that is maintained in all plant and animal cells containing mitochondria and chloroplasts, which are considered to represent ancient bacteria originally engulfed by ancient eukaryotic unicells (Zhao et al.). This viewpoint considerably opens our perspective of pineal melatonin in vertebrates and poses the question of the function(s) and putative molecular targets of melatonin in early evolution.

The issue of melatonin targets is taken up by Dr. Jockers and colleagues who summarize and comment on the high number, more than 15, of reported melatonin targets (Liu et al.). Among them those with the highest affinity for melatonin are those found in vertebrates, the G protein-coupled MT_1_ and MT_2_ receptors (1). The affinity of melatonin for these receptors (~0.1 nM) matches physiological circulating melatonin levels of 5 to 150 pg/ml (~0.65 nM). Further receptors, enzymes, pores, transporters, etc., have been suggested to interact with melatonin at higher (up to millimolar) concentrations (Liu et al.). The chemical properties of melatonin itself as a free radical scavenger in *in vitro* assays hint to additional non-receptor mediated properties of melatonin as a cellular antioxidant (Zhao et al.).

By activating MT_1_ and MT_2_ receptors, melatonin entrains circadian rhythms, regulates seasonal reproduction and retinal physiology, and initiates sleep, the latter discussed in the present Research Topic by Drs. Gobbi and Comai (Gobbi and Comai). Non-selective agonists targeting MT_1_ and MT_2_ receptors like Ramelteon are already marketed for insomnia. The latest pharmacological and knockout studies in mice indicate that subtype selective agonists might be even a better therapeutic choice as MT_1_ and MT_2_ receptors have opposing functions on sleep regulation.

Melatonin’s metabolic effects are increasingly recognized and summarized here by Dr. Tosini and colleagues, who made important contributions to the field by studying receptor knockout mouse models (Owino et al.). Central melatonin receptors in the SCN might be important metabolic regulators as the dysregulation of the circadian master clock in these brain nuclei contribute to metabolic diseases like type 2 diabetes mellitus (2). Melatonin receptors are also found in peripheral tissues such as the liver, muscle, and pancreas where they participate in glucose homeostasis and body weight regulation. The translation of these animal studies into humans remains to be shown. The association of genetic variants of the *MTNR1B* gene, encoding the MT_2_ receptor, with risk of T2DM argues in this direction.

The role of melatonin in reproduction is well established in seasonal breeders. In humans, which are not seasonal, the situation is less clear as summarized by Dr. Olcese, a pioneer in this field. In his article (Olcese), he discusses controversial findings on melatonin and puberty, the entraining role of circulating maternal melatonin on the fetus and its role in oogenesis. Potential therapeutic applications in infertility and melatonin’s synergism with oxytocin in inducing labor are also discussed.

Neurodegenerative diseases such as Parkinson and Alzheimer disease represent a challenge for our aging society. Dr. Cardinali, a pioneer of melatonin biology and its clinical translation, advocates in his article for the supplementation of the age-related decline of melatonin levels in the prevention of neurodegenerative diseases (Cardinali). Beneficial effects of melatonin include its hypnotic, chronobiotic, and cytoprotective properties to improve sleep latency and alleviate sundowning, to stabilize biological rhythms and to limit to a low degree inflammatory damage and neuronal death.

The last word of this Research Topic is due to Dr. Arendt, the “Grande Dame” in the melatonin field. By studying free-running blind subjects, she demonstrated the entrainment effect of exogenous melatonin in humans among many other finding (Arendt). Concerning the function of endogenous melatonin, she advocates that melatonin acts as a brake on abrupt short-term changes of circadian phase by maintaining the circadian status quo. Abrupt changes in the light-dark cycle are experienced by shift workers and time zone travelers and the list of associated risks is substantial (increased rate of accidents, lowered alertness and performance, gut problems, increased risk for metabolic diseases, etc.).

In conclusion, the contributions found in the Research Topic on “Melatonin in Health and Disease” illustrate the power and promises—but also the questions and limitations—in the melatonin field as seen by the experts. The future goal for all remains the same: “… to define the known, to evaluate the novel, and ultimately to inspire unknown future research into this fascinating, ancient molecule.”

## Author Contributions

Both authors contributed to the writing. All authors contributed to the article and approved the submitted version.

## Conflict of Interest

The authors declare that the research was conducted in the absence of any commercial or financial relationships that could be construed as a potential conflict of interest.
